# Crystal structure and Hirshfeld surface analysis of 2-bromo­ethyl­ammonium bromide – a possible side product upon synthesis of hybrid perovskites

**DOI:** 10.1107/S2056989024005619

**Published:** 2024-06-18

**Authors:** Oleksandr A. Semenikhin, Sergiu Shova, Irina A. Golenya, Dina D. Naumova, Il’ya A. Gural’skiy

**Affiliations:** a Department of Chemistry, Taras Shevchenko National University of Kyiv, Volodymyrska St. 64, Kyiv 01601, Ukraine; b Department of Inorganic Polymers, Petru Poni Institute of Macromolecular Chemistry, Aleea Grigore Ghica Voda 41-A, Iasi 700487, Romania; Vienna University of Technology, Austria

**Keywords:** crystal structure, Hirshfeld surface analysis, 2-bromo­ethyl­amine hydro­bromide, hybrid perovskite

## Abstract

This study presents the synthesis, characterization and Hirshfeld surface analysis of a small organic ammonium salt, C_2_H_7_BrN^+^·Br^−^. Small cations like the one in the title compound are considered promising components of hybrid perovskites, crucial for optoelectronic and photovoltaic applications. While the incorporation of this organic cation into various hybrid perovskite structures has been explored, its halide salt counterpart remains largely uninvestigated. The obtained structural results are valuable for the synthesis and phase analysis of hybrid perovskites. The title compound crystallizes in the solvent-free form in the centrosymmetric monoclinic space group *P*2_1_/*c*, featuring one organic cation and one bromide anion in its asymmetric unit, with a torsion angle of −64.8 (2)° between the ammonium group and the bromine substituent, positioned in a *gauche* conformation. The crystal packing is predominantly governed by Br⋯H inter­actions, which constitute 62.6% of the overall close atom contacts.

## Abstract 2

This study describes the synthesis, characterization, and Hirshfeld surface analysis of a small organic ammonium bromide salt. The analysis reveals a torsion angle of −64.8 (2)° between the ammonium group and the bromine substituent.

## 1. Chemical context

Hybrid perovskites have emerged as a class of highly promising compounds for a wide array of applications in optoelectronics and photovoltaics due to their semiconducting properties. Among these, perovskites with a tri-periodic arrangement have garnered significant attention owing to their optimal bandgap width (Dey *et al.*, 2021 ▸; Liu *et al.*, 2021 ▸; Hassan *et al.*, 2021 ▸; Yoo *et al.*, 2021 ▸). Notably, the aziridinium cation (AzrH) has recently been shown to support such perovskite structures. In the form (AzrH)*BX*_3_ (*B* = Pb, Sn; *X* = Br, I; Petrosova *et al.*, 2022 ▸; Kucheriv *et al.*, 2023 ▸), these perovskites display promising physical properties (Mączka *et al.*, 2023 ▸; Stefańska *et al.*, 2022 ▸), and nanomaterials based on them offer potential for various applications (Semenikhin *et al.*, 2023 ▸; Bodnarchuk *et al.*, 2024 ▸).

The high reactivity of aziridine poses a synthetic challenge as it can undergo ring-opening in acidic environments, leading to the formation of perovskites with low periodicity such as (*X*(CH_2_)_2_NH_3_)_2*n*_(*BX*)_4*n*_ (*B* = Pb, Sn; *X* = Br, I; Skorokhod *et al.*, 2023 ▸; Song *et al.*, 2022 ▸; Sourisseau *et al.*, 2007 ▸; Lemmerer & Billing, 2010 ▸) and 2-bromo­ethyl­ammonium bromide as a side product. However, these perovskite materials also often manifest physical properties that are as well worth exploring.

In this study, we present the crystal structure analysis and Hirshfeld surface analysis of an organic–inorganic hybrid salt, C_2_H_7_BrN^+^Br^−^. While this organic cation has previously been incorporated into various hybrid perovskite structures, its halide salt counterpart remains unexplored, representing a significant gap in analysis of these materials. Knowledge of its structure is also important for the phase analysis of studied aziridinium-based materials.
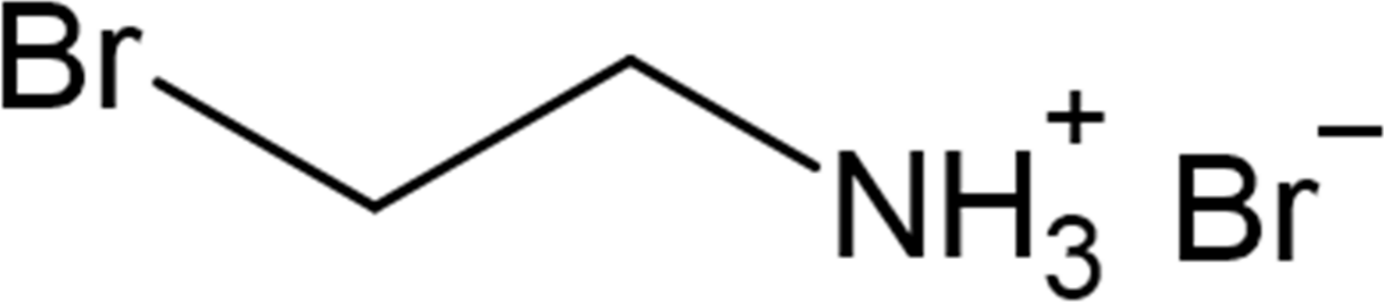


## 2. Structural commentary

**Figure 1:**
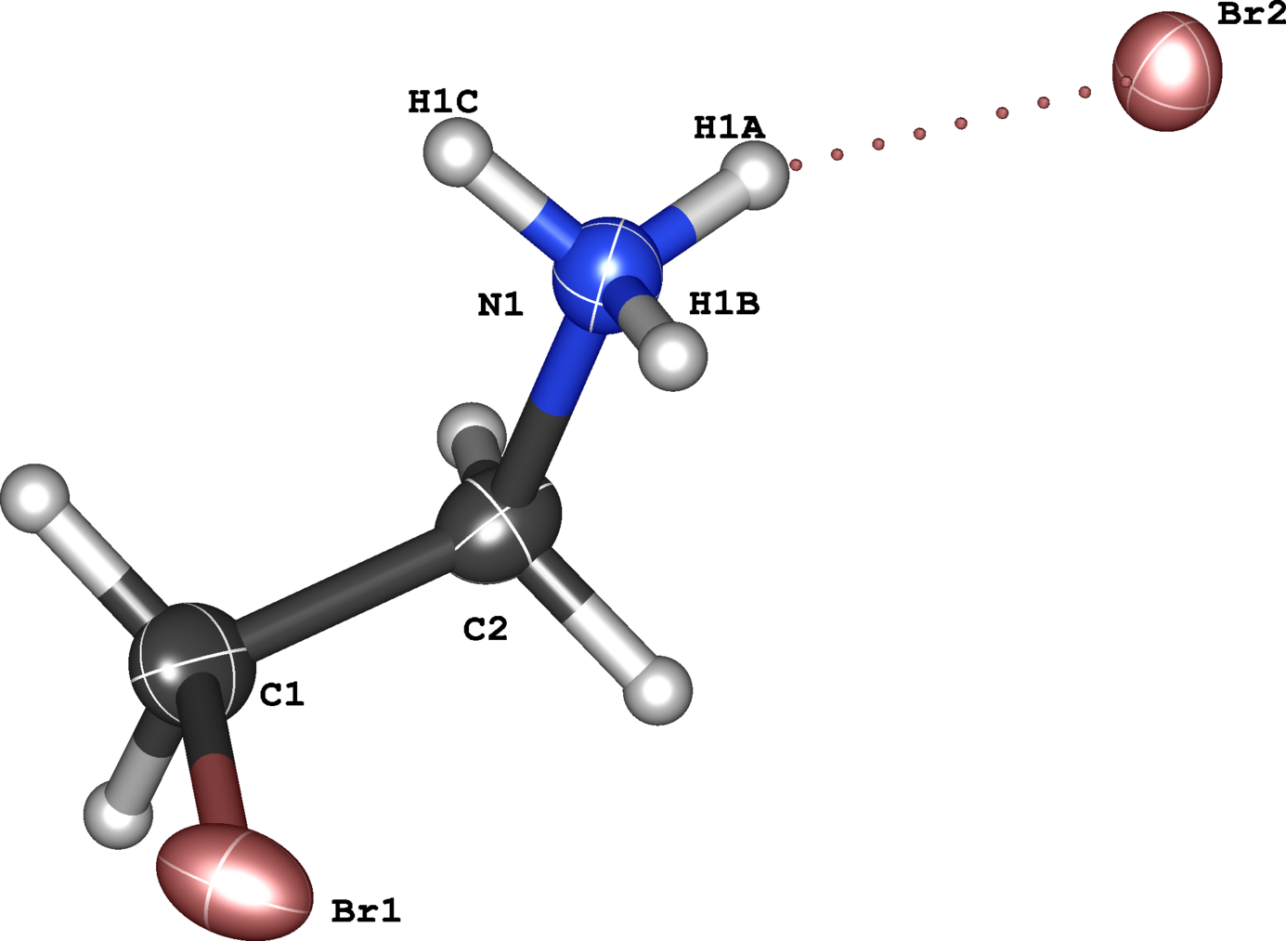
The asymmetric unit of 2-bromo­ethyl­ammonium bromide with displacement ellipsoids drawn at the 50% probability level. The dotted line represents the hydrogen bond between the cation and anion.

The title compound crystallizes in a solvent-free form and consists of one organic cation and one bromide anion in the asymmetric unit (Fig. 1 ▸). The backbone of the cation, N1, C2, C1, Br1, has a torsion angle of −64.8 (2)°, with the atoms positioned in a *gauche* conformation. The N1—C2 and C1—C2 bonds have lengths of 1.480 (3) and 1.513 (4) Å, respectively. These values are typical for protonated alk­ylamines and consistent with previous reports (Ishida, 2000 ▸). The C1—Br1 length is 1.953 (3) Å, which is also a typical value for C—*X* length in alkyl halides (Allen *et al.*, 1987 ▸).

## 3. Inter­molecular features

**Figure 2:**
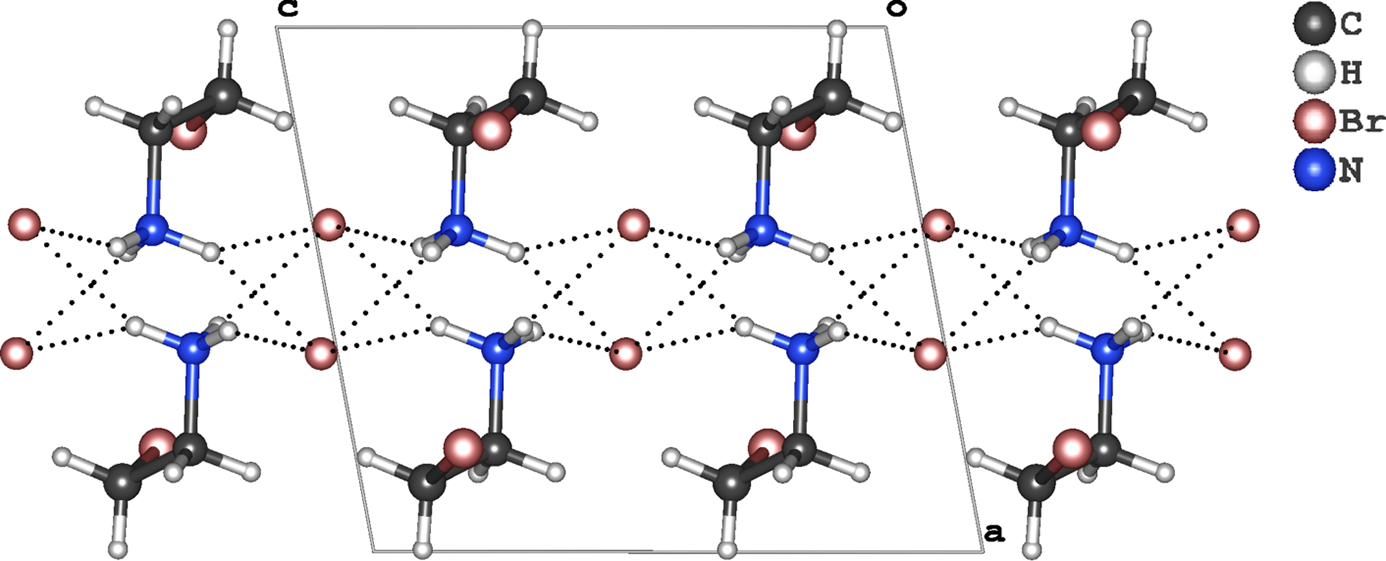
Projection of the crystal structure along the *b* axis, showing the hydrogen-bonding inter­actions with the anion being an acceptor of four N—H⋯Br hydrogen bonds.

**Figure 3:**
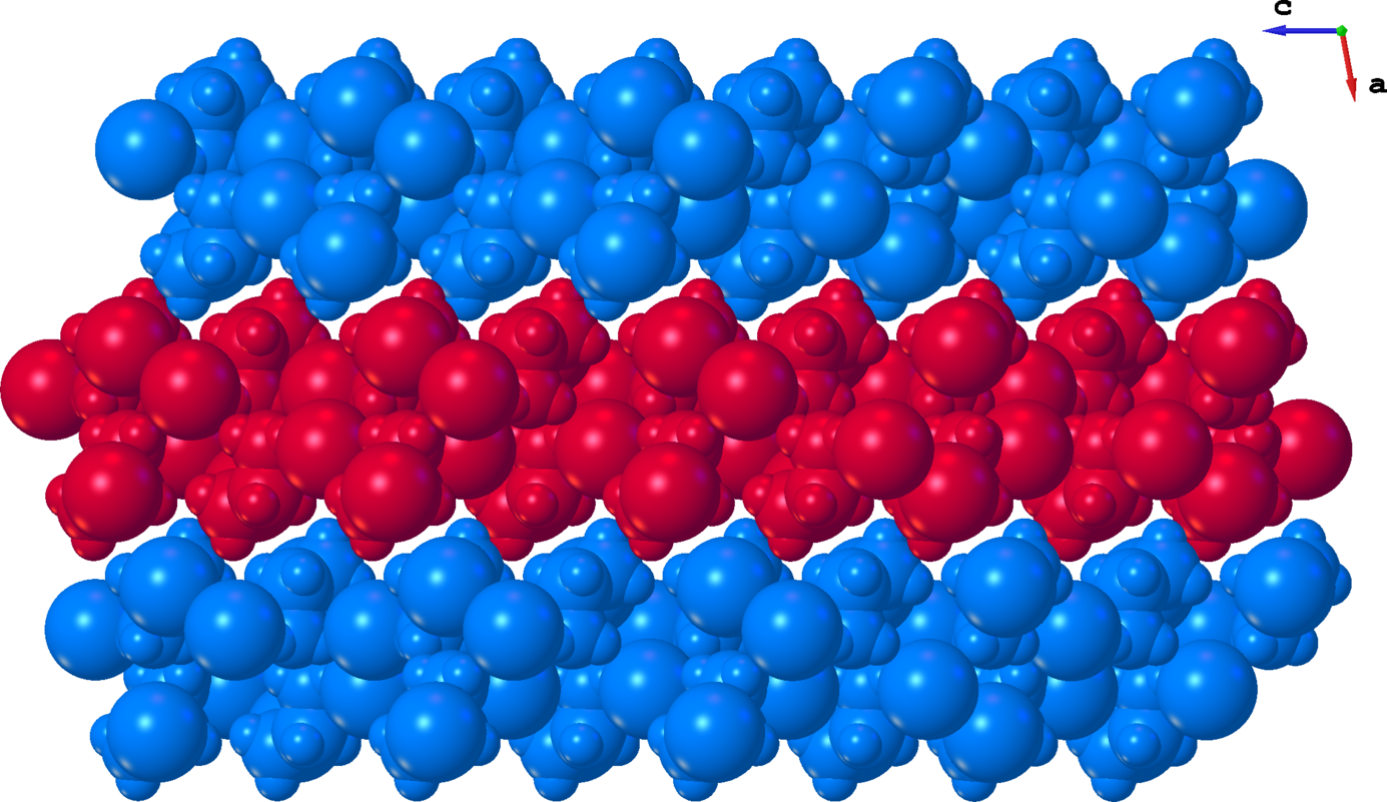
Space-filling model of the title compound showing the organization into double layers extending parallel to (100).

Fig. 2 ▸ shows a view of the structure along the *b* axis, which illustrates the inter­molecular organization through N—H⋯Br hydrogen bonds, revealing that each bromide anion is the acceptor of four contacts with NH_3_^+^ groups. Corresponding numerical data are given in Table 1 ▸. Our analysis uncovered different patterns of hydrogen-bonding inter­actions. Specifically, N1—H1*A*⋯Br2 and N1—H1*B*⋯Br2^i^ [symmetry code: (i) −*x* + 1, −*y* + 1, −*z* + 1] inter­actions demonstrate typical classical behavior, with angles of 156.1° and 156.2°, and *D*⋯*A* distances of 3.3010 (19) Å and 3.381 (2) Å, respectively. In contrast, N1—H1*C*⋯Br2^ii^ and N1—H1*C*⋯Br2^iii^ [symmetry codes: (ii) *x*, −*y* + 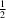, *z* + 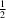; (iii) −*x* + 1, *y* + 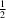, −*z* + 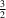] contacts exhibit weaker inter­actions, with longer *D*⋯*A* distances of 3.3904 (19) and 3.4292 (18) Å, and angles of 125.1° and 140.3°. Fig. 3 ▸ shows that the arrangement of cations and anions leads to the formation of double layers.

## 4. Hirshfeld analysis

**Figure 4:**
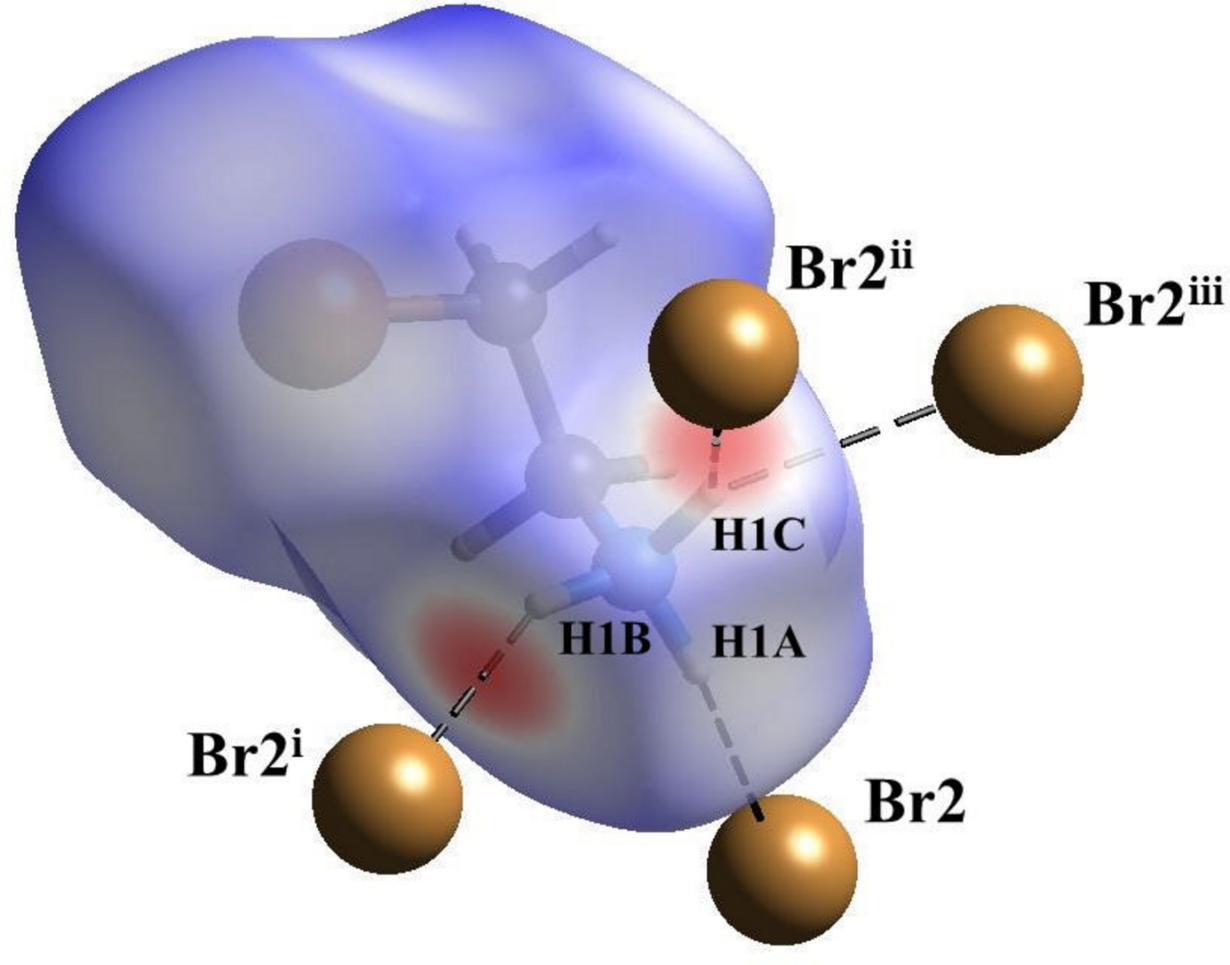
Three-dimensional model of the Hirshfeld surface for 2-bromo­ethyl­ammonium bromide mapped over *d*_norm_, representing strong inter­molecular inter­actions. [Symmetry codes: (i) −*x* + 1, −*y* + 1, −*z* + 1; (ii) *x*, −*y* + 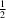, *z* + 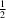; (iii) −*x* + 1, *y* + 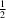, −*z* + 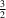.]

**Figure 5:**
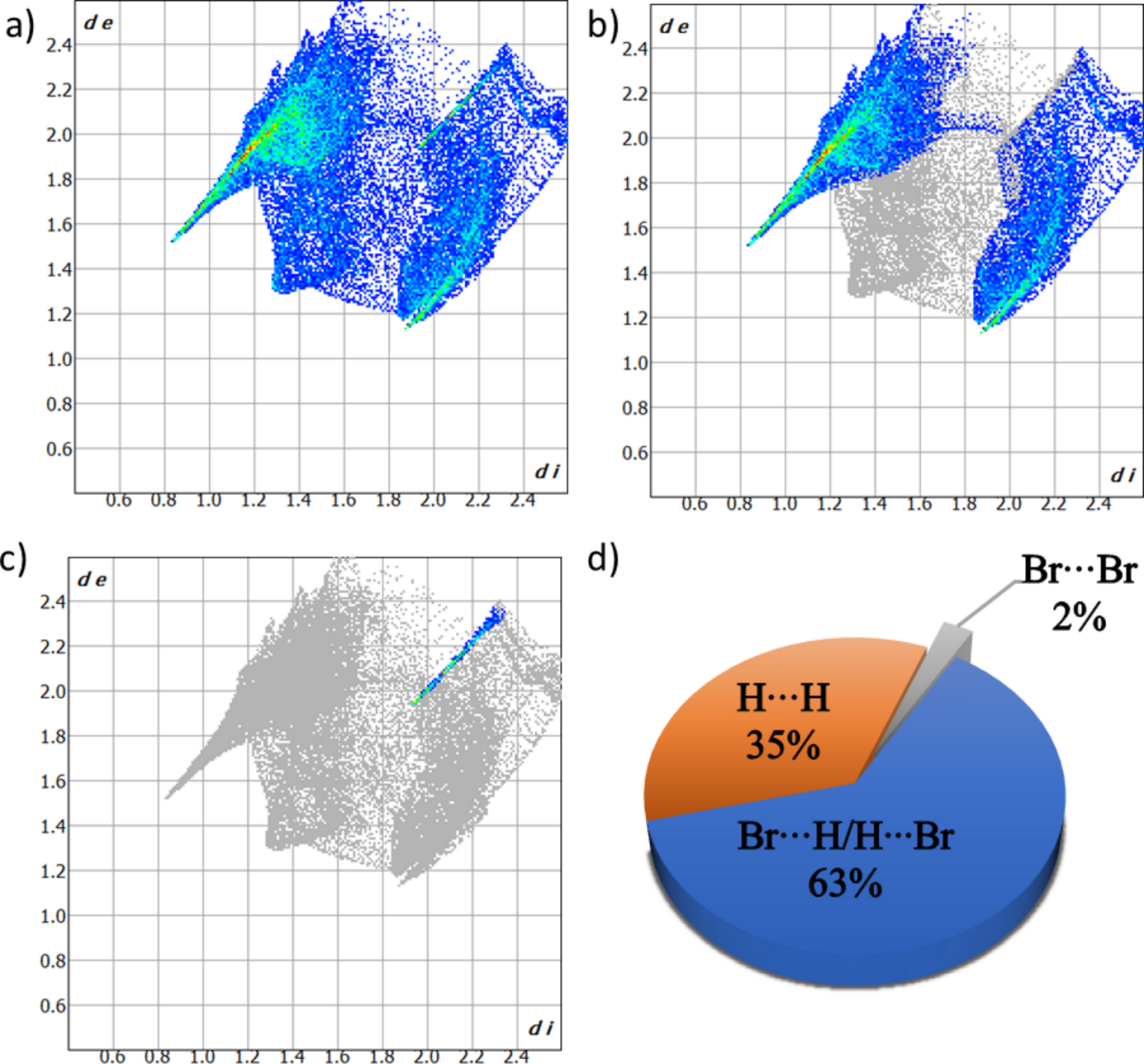
Two-dimensional fingerprint plots of 2-bromo­ethyl­ammonium bromide showing (*a*) all inter­actions, (*b*) Br⋯H/H⋯Br and (*c*) Br⋯Br inter­actions (*d*_i_ and *d*_e_ are the closest inter­nal and external distances in Å on the Hirshfeld surface) and (*d*) their percentage contributions.

The inter­molecular inter­actions in 2-bromo­ethyl­ammonium bromide were analyzed using Hirshfeld surface calculations, employing *CrystalExplorer (*Spackman *et al.*, 2021 ▸). Results are plotted over the *d*_norm_ range between −0.4077 and +1.2052 a.u. (Spackman & Jayatilaka, 2009 ▸). A three-dimensional model of the Hirshfeld surface (Fig. 4 ▸) highlights strong Br⋯H/H⋯Br contacts, exhibiting a cation volume of 103.45 Å, a surface area of 119.7 Å, a globularity of 0.890, and an asphericity of 0.059. Additionally, two-dimensional fingerprint plots were generated, illustrating all specific inter­molecular contacts (McKinnon *et al.*, 2007 ▸). Fig. 5 ▸ shows Br⋯H/H⋯Br, Br⋯Br inter­actions, and all inter­actions present in the structure with meaningful inter­molecular contacts. In the crystal packing, Br⋯H inter­actions predominate, constituting 62.6% of the overall close atom contacts, while Br⋯Br inter­actions contribute with 2.6%, and H⋯H contacts account for 34.8%, indicating no additional inter­actions involving the heteroatoms.

## 5. Synthesis and crystallization

All chemicals were purchased from Enamine Ltd (Kyiv, Ukraine) and used without any further purification. Aziridine (258.4 µl, 5 mmol) was added dropwise under stirring to 2 ml of conc. HBr, gradually heated to 353 K until water evaporation occurred and colorless crystals formed. The obtained crystals were left under Paratone(R) oil until the X-ray measurement.

## 6. Refinement

Crystal data, data collection and structure refinement details are summarized in Table 2 ▸. Hydrogen atoms were placed at calculated positions with *U*_iso_(H) = 1.2*U*_eq_(C,N). Hydrogens atom of CH_2_ group were included in idealized positions (C—H = 0.99 Å).

## Supplementary Material

Crystal structure: contains datablock(s) I. DOI: 10.1107/S2056989024005619/wm5723sup1.cif

Structure factors: contains datablock(s) I. DOI: 10.1107/S2056989024005619/wm5723Isup2.hkl

Supporting information file. DOI: 10.1107/S2056989024005619/wm5723Isup3.cml

CCDC reference: 2362029

Additional supporting information:  crystallographic information; 3D view; checkCIF report

## Figures and Tables

**Table 1:** Hydrogen-bond geometry (Å, °)

*D*—H⋯*A*	*D*—H	H⋯*A*	*D*⋯*A*	*D*—H⋯*A*
N1—H1*A*⋯Br2	0.85	2.51	3.3010 (19)	156
N1—H1*B*⋯Br2^i^	0.85	2.59	3.381 (2)	156
N1—H1*C*⋯Br2^ii^	0.85	2.83	3.3904 (19)	125
N1—H1*C*⋯Br2^iii^	0.85	2.73	3.4292 (18)	140

Symmetry codes: (i) 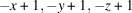; (ii) 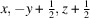; (iii) 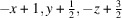.

**Table 2:** Experimental details

Crystal data	
Chemical formula	C_2_H_7_BrN^+^·Br^−^
*M*_r_	204.91
Crystal system, space group	Monoclinic, *P*2_1_/*c*
Temperature (K)	200
*a*, *b*, *c* (Å)	7.8966 (4), 8.3394 (4), 9.0089 (4)
β (°)	100.546 (5)
*V* (Å^3^)	583.24 (5)
*Z*	4
Radiation type	Mo *K*α
μ (mm^−1^)	13.75
Crystal size (mm)	0.15 × 0.05 × 0.02

Data collection	
Diffractometer	XtaLAB Synergy, Dualflex, HyPix
Absorption correction	Multi-scan (*CrysAlis PRO*; Rigaku OD, 2024 ▸)
*T*_min_, *T*_max_	0.451, 1.000
No. of measured, independent and observed [*I* > 2σ(*I*)] reflections	4504, 1453, 1242
*R*_int_	0.024
(sin θ/λ)_max_ (Å^−1^)	0.709

Refinement	
*R*[*F*^2^ > 2σ(*F*^2^)], *wR*(*F*^2^), *S*	0.024, 0.054, 1.04
No. of reflections	1453
No. of parameters	49
H-atom treatment	H atoms treated by a mixture of independent and constrained refinement
Δρ_max_, Δρ_min_ (e Å^−3^)	0.56, −0.47

Computer programs: *CrysAlis PRO* (Rigaku OD, 2024 ▸), *SHELXT* (Sheldrick, 2015*a* ▸), *SHELXL* (Sheldrick, 2015*b* ▸) and *OLEX2* (Dolomanov *et al.*, 2009 ▸).

## supplementary crystallographic information

### 2-Bromoethylammonium bromide Crystal data

**Table:**

C_2_H_7_BrN^+^·Br^−^	*F*(000) = 384
*M**_r_* = 204.91	*D*_x_ = 2.334 Mg m^−^^3^
Monoclinic, *P*2_1_/*c*	Mo *K*α radiation, λ = 0.71073 Å
*a* = 7.8966 (4) Å	Cell parameters from 2526 reflections
*b* = 8.3394 (4) Å	θ = 2.6–30.0°
*c* = 9.0089 (4) Å	µ = 13.75 mm^−^^1^
β = 100.546 (5)°	*T* = 200 K
*V* = 583.24 (5) Å^3^	Plate, clear intense colourless
*Z* = 4	0.15 × 0.05 × 0.02 mm

### 2-Bromoethylammonium bromide Data collection

**Table:**

XtaLAB Synergy, Dualflex, HyPix diffractometer	1453 independent reflections
Radiation source: micro-focus sealed X-ray tube, PhotonJet (Mo) X-ray Source	1242 reflections with *I* > 2σ(*I*)
Mirror monochromator	*R*_int_ = 0.024
Detector resolution: 10.0000 pixels mm^-1^	θ_max_ = 30.3°, θ_min_ = 2.6°
ω scans	*h* = −10→10
Absorption correction: multi-scan (CrysAlisPro; Rigaku OD, 2024)	*k* = −10→11
*T*_min_ = 0.451, *T*_max_ = 1.000	*l* = −12→12
4504 measured reflections	

### 2-Bromoethylammonium bromide Refinement

**Table:**

Refinement on *F*^2^	Hydrogen site location: inferred from neighbouring sites
Least-squares matrix: full	H atoms treated by a mixture of independent and constrained refinement
*R*[*F*^2^ > 2σ(*F*^2^)] = 0.024	*w* = 1/[σ^2^(*F*_o_^2^) + (0.028*P*)^2^] where *P* = (*F*_o_^2^ + 2*F*_c_^2^)/3
*wR*(*F*^2^) = 0.054	(Δ/σ)_max_ = 0.001
*S* = 1.04	Δρ_max_ = 0.56 e Å^−^^3^
1453 reflections	Δρ_min_ = −0.47 e Å^−^^3^
49 parameters	Extinction correction: SHELXL (Sheldrick, 2015b), Fc^*^=kFc[1+0.001xFc^2^λ^3^/sin(2θ)]^-1/4^
0 restraints	Extinction coefficient: 0.0055 (7)
Primary atom site location: dual	

### 2-Bromoethylammonium bromide Special details

**Table:**

Geometry. All esds (except the esd in the dihedral angle between two l.s. planes) are estimated using the full covariance matrix. The cell esds are taken into account individually in the estimation of esds in distances, angles and torsion angles; correlations between esds in cell parameters are only used when they are defined by crystal symmetry. An approximate (isotropic) treatment of cell esds is used for estimating esds involving l.s. planes.

### 2-Bromoethylammonium bromide Fractional atomic coordinates and isotropic or equivalent isotropic displacement parameters (Å^2^)

**Table:**

	*x*	*y*	*z*	*U*_iso_*/*U*_eq_	
Br2	0.62288 (3)	0.21658 (3)	0.52611 (2)	0.02586 (10)	
Br1	0.80287 (4)	0.89002 (3)	0.82138 (3)	0.03709 (11)	
N1	0.6130 (3)	0.5414 (2)	0.7338 (2)	0.0252 (4)	
H1A	0.5825 (5)	0.4579 (15)	0.6819 (15)	0.030*	
H1B	0.5747 (6)	0.6239 (14)	0.6835 (16)	0.030*	
H1C	0.5728 (7)	0.5380 (17)	0.8149 (12)	0.030*	
C2	0.8034 (3)	0.5490 (3)	0.7706 (3)	0.0265 (5)	
H2A	0.848456	0.575438	0.678011	0.032*	
H2B	0.848904	0.442408	0.806124	0.032*	
C1	0.8670 (4)	0.6727 (3)	0.8908 (3)	0.0333 (6)	
H1D	0.993935	0.665423	0.918954	0.040*	
H1E	0.817760	0.649328	0.981906	0.040*	

### 2-Bromoethylammonium bromide Atomic displacement parameters (Å^2^)

**Table:**

	*U*^11^	*U*^22^	*U*^33^	*U*^12^	*U*^13^	*U*^23^
Br2	0.03128 (17)	0.02218 (16)	0.02435 (15)	0.00045 (10)	0.00574 (11)	−0.00179 (9)
Br1	0.02685 (18)	0.03231 (18)	0.0526 (2)	−0.00574 (11)	0.00868 (13)	−0.00512 (11)
N1	0.0289 (12)	0.0227 (10)	0.0238 (10)	0.0008 (9)	0.0048 (9)	0.0001 (8)
C2	0.0235 (14)	0.0286 (13)	0.0286 (12)	0.0067 (11)	0.0076 (10)	0.0059 (10)
C1	0.0256 (15)	0.0420 (15)	0.0294 (13)	−0.0019 (13)	−0.0023 (11)	0.0071 (11)

### 2-Bromoethylammonium bromide Geometric parameters (Å, º)

**Table:**

Br1—C1	1.953 (3)	C2—H2A	0.9900
N1—H1A	0.849 (12)	C2—H2B	0.9900
N1—H1B	0.849 (12)	C2—C1	1.513 (4)
N1—H1C	0.849 (12)	C1—H1D	0.9900
N1—C2	1.480 (3)	C1—H1E	0.9900
			
H1A—N1—H1B	109.5	H2A—C2—H2B	107.9
H1A—N1—H1C	109.5	C1—C2—H2A	109.1
H1B—N1—H1C	109.5	C1—C2—H2B	109.1
C2—N1—H1A	109.5	Br1—C1—H1D	109.3
C2—N1—H1B	109.5	Br1—C1—H1E	109.3
C2—N1—H1C	109.5	C2—C1—Br1	111.77 (17)
N1—C2—H2A	109.1	C2—C1—H1D	109.3
N1—C2—H2B	109.1	C2—C1—H1E	109.3
N1—C2—C1	112.4 (2)	H1D—C1—H1E	107.9
			
N1—C2—C1—Br1	−64.8 (2)		

### 2-Bromoethylammonium bromide Hydrogen-bond geometry (Å, º)

**Table:**

*D*—H···*A*	*D*—H	H···*A*	*D*···*A*	*D*—H···*A*
N1—H1*A*···Br2	0.85	2.51	3.3010 (19)	156
N1—H1*B*···Br2^i^	0.85	2.59	3.381 (2)	156
N1—H1*C*···Br2^ii^	0.85	2.83	3.3904 (19)	125
N1—H1*C*···Br2^iii^	0.85	2.73	3.4292 (18)	140

Symmetry codes: (i) −*x*+1, −*y*+1, −*z*+1; (ii) *x*, −*y*+1/2, *z*+1/2; (iii) −*x*+1, *y*+1/2, −*z*+3/2.
